# From Mini to Micro Scale—Feasibility of Raman Spectroscopy as a Process Analytical Tool (PAT)

**DOI:** 10.3390/pharmaceutics3040723

**Published:** 2011-10-14

**Authors:** Markus Wirges, Joshua Müller, Péter Kása, Géza Regdon, Klára Pintye-Hódi, Klaus Knop, Peter Kleinebudde

**Affiliations:** 1 Institute of Pharmaceutics and Biopharmaceutics, Heinrich-Heine-University, Universitätsstr. 1, D-40225 Düsseldorf, Germany; E-Mails: markus.wirges@hhu.de (M.W.); klaus.knop@hhu.de (K.K.); 2 Hüttlin GmbH, Hohe-Flum-Strasse 42, D-79650 Schopfheim, Germany; E-Mail: joshua.mueller@oystar-group.de; 3 Department of Pharmaceutical Technology, University of Szeged, Eotvos u. 6, H-6720 Szeged, Hungary; E-Mails: p.kasa@pharm.u-szeged.hu (P.K.); geza.regdon@pharm.u-szeged.hu (G.R.); klara.hodi@pharm.u-szeged.hu (K.P.-H.)

**Keywords:** mini scale, micro scale, active coating, Raman spectroscopy, PAT tool

## Abstract

**Background:**

Active coating is an important unit operation in the pharmaceutical industry. The quality, stability, safety and performance of the final product largely depend on the amount and uniformity of coating applied. Active coating is challenging regarding the total amount of coating and its uniformity. Consequently, there is a strong demand for tools, which are able to monitor and determine the endpoint of a coating operation. In previous work, it was shown that Raman spectroscopy is an appropriate process analytical tool (PAT) to monitor an active spray coating process in a pan coater [1]. Using a multivariate model (Partial Least Squares—PLS) the Raman spectral data could be correlated with the coated amount of the API diprophylline. While the multivariate model was shown to be valid for the process in a mini scale pan coater (batch size: 3.5 kg cores), the aim of the present work was to prove the robustness of the model by transferring the results to tablets coated in a micro scale pan coater (0.5 kg).

**Method:**

Coating experiments were performed in both, a mini scale and a micro scale pan coater. The model drug diprophylline was coated on placebo tablets. The multivariate model, established for the process in the mini scale pan coater, was applied to the Raman measurements of tablets coated in the micro scale coater for six different coating levels. Then, the amount of coating, which was predicted by the model, was compared with reference measurements using UV spectroscopy.

**Results:**

For all six coating levels the predicted coating amount was equal to the amounts obtained by UV spectroscopy within the statistical error. Thus, it was possible to predict the total coating amount with an error smaller than 3.6%. The root mean squares of errors for calibration and prediction (root mean square of errors for calibration and prediction—RMSEC and RMSEP) were 0.335 mg and 0.392 mg, respectively, which means that the predictive power of the model is not dependent on the scale or the equipment.

**Conclusion:**

The scale-down experiment showed that it was possible to transfer the PLS model developed on a mini scale coater to a micro scale coater.

## 1. Introduction

Pan coating, as an important unit operation for the formulation of solid oral dosage forms, can crucially influence the performance of a medicinal product. Film coatings are applied to pharmaceutical solid dosage forms for a number of purposes and may be categorized on the basis of their influence on drug release: Immediate release coatings, which are often used to improve product stability, to facilitate product identification and to improve in product organoleptic characteristics. Modified release coatings can be subdivided into delayed-release (e.g., enteric) coatings and extended-release (sustained-release) coatings and are used to achieve a desired therapeutic effect. Active coating (where the active ingredient is coated onto the core) can be classified as an immediate release coating and upgrades an oral dosage form in terms of the drug release profile. Fixed dose combinations in the core and the coating, respectively, are a typical field of application. Active coating is challenging with regard to the total amount of coating and its uniformity. Consequently, there is a strong demand for tools that are able to monitor and determine the endpoint of a coating operation.

In previous work, Romero-Torres *et al.* [2,3] and El Hagrasy *et al.* [4,5] implemented Raman spectroscopy as a PAT tool for coating processes by correlating Raman spectra of coated tablets with coating time, respectively Raman spectra of coated tablets with the average weight gain of tablets. Moreover, Raman spectroscopy had also been previously found to be suitable for the calibration of the coating thickness [6]. For the first time Müller *et al.* [1] focused on the determination of the amount and uniformity of active coating. Using a multivariate model (Partial Least Squares—PLS), the Raman spectral data could be correlated with the coated amount of the API diprophylline. While the multivariate model has been shown to be valid for the process in a mini scale pan coater (batch size: 3.5 kg cores), the aim of the present work was to prove the robustness of the model by transferring the results to tablets coated in a micro scale pan coater (0.5 kg).

## 2. Experimental Section

### 2.1. Materials

#### 2.1.1. Drug

The water-soluble caffeine derivative diprophylline (BASF, Ludwigshafen, Germany) was used for the active coating as model drug because it is detectable well by both, Raman and UV spectroscopy.

#### 2.1.2. Tablets

Lactose monohydrate (Tablettose^®^ 80, Flowlac^®^ 100; Meggle, Wasserburg, Germany), microcrystalline cellulose (Avicel^®^ PH 102; FMC International, Little Island Co., Cork, Ireland) and magnesium stearate (Welding, Hamburg, Germany) were used as excipients of the core.

#### 2.1.3. Coating Solution

The aqueous coating solution was composed of hydroxypropyl methylcellulose (HPMC, Walocel^®^ HM5 PA2910; Wolff Cellulosics, Walsrode, Germany), polyethylene glycol 1500 (Clariant GmbH, Frankfurt am Main, Germany) and diprophylline (BASF; Ludwigshafen, Germany).

### 2.2. Methods

#### 2.2.1. Placebo Cores

The tablet cores were biconvex (4 mm in height, 8 mm in diameter, average weight: 200 mg) and were composed of 49.75% (w/w) lactose monohydrate (Tablettose^®^ 80), 49.75% (w/w) microcrystalline cellulose, and 0.5% (w/w) magnesium stearate.

#### 2.2.2. Coating Solution

An aqueous-based coating solution was prepared, consisting of 12% w/w diprophylline, 6% w/w hydroxypropyl methylcellulose and 2% w/w polyethylene glycol 1500. The polyethylene glycol and diprophylline were added to 2/3 of distilled water. After the API and polyethylene glycol were dissolved, the hydroxypropyl methylcellulose, which was dispersed in 1/3 of distilled water of 90 °C, was slowly added. Then the solution was mixed for 20 minutes to disperse the polymer. The solution was allowed to rest for at least 12 h to hydrate the polymer before use.

#### 2.2.3. Tablet Coating

Both, lab and micro-scale batches of tablet cores were coated with the same film coating formulation. The lab-scale batch was coated using a BFC 5, Bohle Film Coater (L.B. Bohle, Ennigerloh, Germany). A Pro-C-epT 4M8 Film Coater (Pro-C-epT, Zelzate, Belgium) was used to coat the micro-scale batch (Table 1).

The process parameters are shown in Tables 2 and 3.

Over a period of 150 min, 20 tablets at a time were collected every 30 minutes. At the end of the micro scale coating process an average of (11 ± 0.5) mg diprophylline as measured by UV spectroscopy was coated onto each tablet.

#### 2.2.4. Raman Measurements and Calculations

For the Raman measurements a RamanRXN2 analyzer of Kaiser Optical Systems (Ann Arbor, USA) with a laser wavelength of 785 nm was used. The spectrometer was equipped with a non-contact optic sampling device (PhAT probe). The excitation laser (785 nm diode laser) was introduced and magnified to form a circular illumination area of 6 mm diameter (area: 28.3 mm^2^) to cover a large sample area, which improves the reliability and reproducibility of Raman measurements.

Data collection and all the calculations including baseline correction (Standard Normal Variate—SNV), intensity normalization and partial least squares (PLS) regression, were done using icRaman^®^ data collection software package (Kaiser Optical Systems, Ann Arbor, USA), SIMCA-P+ 12.0.1^®^ (Umetrics, Umea, Sweden), the Matlab^®^ software package (version 6.5, The MathWorks, Inc., Natick, MA, USA), and Excel^®^ (version 2007, Microsoft Corporation).

#### 2.2.5. Reference Analysis

To determine the amount of coated diprophylline an UV spectroscopic method was applied (Lambda-2, Perkin-Elmer, Überlingen, Germany). The calibration covered a range between 1.00 mg/500 mL and 20.03 mg/500 mL. Three measurements at 273 nm were performed for each calibration level and for each tablet after being dissolved in distilled water.

#### 2.2.6. Calibration and Validation Development

Tablets were collected from the mini scale coater at different stages of coating (tablet sets from 0–12.7 mg diprophylline). For the off-line quantitative calibration development these tablets (*n* = 42) were measured by Raman spectroscopy with a scanning time of 15 seconds for each tablet. Then, the multivariate model was built up with this data set. As reference analytical method, UV spectroscopy was applied to the same tablets, in order to obtain the amount of coated diprophylline.

The same multivariate model, derived from the coating process at mini scale coater, was applied to tablets coated in the micro scale coater. Again, tablets were collected at six coating stages on the micro scale run (tablet sets from 0–11.8 mg diprophylline). Then, the amount of coating, which was predicted by the model, was compared with measurements using UV spectroscopy.

## 3. Results and Discussion

A Partial Least Squares (PLS) predictive model was constructed using the Standard Normal Variate (SNV) spectral data from the tablet sets with 0 to 12.7 mg diprophylline (Table 4).

**Figure 1.**
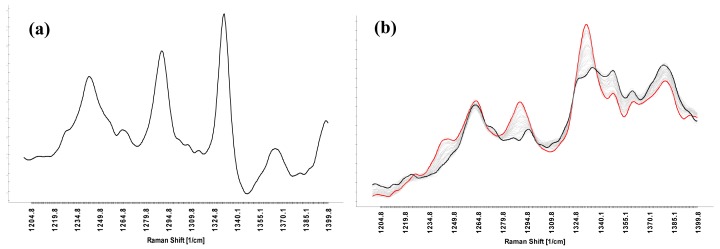
(**a**) p-Loadings of PC1 in comparison to (**b**) Raman spectrum of the tablets in dependence on the coating level.

Figure 1(b) shows the selected wavenumber range (1200–1400 cm^−1^) the multivariate model (mini scale) is based on. Both peaks (1290 and 1330 cm^−1^), which can be assigned to diprophylline (CN-stretch and imidazole ring stretch), gain intensity with coating time, reflecting the increasing amount of diprophylline. Best predictive results for this model were obtained by using three principal components, which explain 94.4% of the X-Variance (spectral data) and 99.4% of the Y-Variance (coated diprophylline amount).

**Figure 2.**
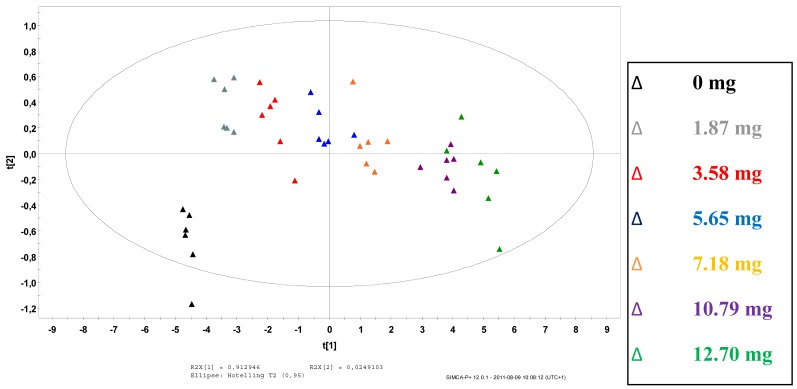
Principal component analysis (PCA) scores plot obtained from Standard Normal Variate (SNV) preprocessed Raman spectra (API content; *n* = 6; mean).

Principal component analysis (PCA) of the Raman spectra acquired during the coating operation was performed to discriminate tablets by increasing mass of coating materials. PCA scores on the first principal component (PC1) are able to order the samples with respect to the theoretical amount of API in the coating, as illustrated in Figure 2. PC1 explains 91.3% of the variance. Loadings on PC1 mainly correspond to spectral information from the API diprophylline (Figure 1(a)). The construction of PC1 can therefore be explained by the increasing amount of API coated onto the tablet core.

**Figure 3.**
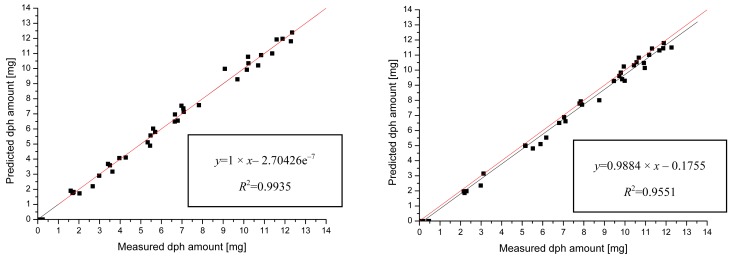
Calibration (left) and validation (right) variance regression model for SNV Raman spectra.

In Figure 3 each data point displays a tablet with a certain amount of diprophylline, which was measured by Raman and UV spectroscopy. On the y-axis, the amount of diprophylline as predicted by the multivariate model derived from Raman measurements is given. The x-axis represents the corresponding data as obtained by the reference measurement (UV spectroscopy). The left panel shows the result of the calibration process for the mini scale coater. The parameter set for the multivariate model of the calibration process was used to analyze the Raman data obtained by measurements on tablets collected at different coating times from the micro scale coater. The results of this validation are shown in the right panel of Figure3. For an ideal fit of the predicted model, one would expect a linear increase with a slope of one (red line). The black line shows the linear fit to the data points. The predicted amount of diprophylline obtained by the multivariate model fits very nicely the real amount of diprophylline measured by UV spectroscopy.

The root mean square of errors for calibration and prediction (RMSEC and RMSEP) are direct estimates of the modeling error and the prediction error, expressed in original measurement units. In this work, the RMSEC of 0.335 mg describes the error of the model built up at the mini scale coater. The RMSEP of 0.392 mg, being almost equal to the RMSEC, shows that the model developed for the mini scale coater was successfully applied to the micro scale coater. Consequently, the predictive power of the multivariate model is not dependent on the scale or the equipment, since the predicted amount of coating was equal to the amount obtained by UV spectroscopy within the statistical error of 3.6% (calculated as RMSEP related to the average coating amount at the end of the coating run).

## 4. Conclusions

In the present work, it was shown that Raman spectroscopy used as a PAT tool could be implemented in different scales of a coating process. Especially, it was demonstrated that multivariate models developed to correlate the API's coating amount to the Raman spectra for a coating process in the mini scale coater could be transferred to micro scale processes. Thus, there is no need to develop a new multivariate model when dealing with scale up, scale down or switching of the equipment. Since this work focused on the robustness of multivariate models regarding off-line measurements, it wouldbe highly desirable for future investigations to deal with the transfer of inline measurements between different scales.

## Figures and Tables

**Table 1.** Coating equipment.

	**Mini Scale**	**Micro Scale**
**Equipment**	BFC5, L.B.B. Bohle Ennigerloh, Germany	4M8, Pro-C-epT Zelzate, Belgium
**Coating pan**	316 mm/356 mm (diameter/length)	203 mm/145 mm (diameter/length)
**Spray nozzle**	970/7-1 S75, Düsen Schlick Untersiemau, Germany	0,71 mm (diameter) round spray
**Batch size**	3.5kg	500g

**Table 2.** Coating parameters BFC 5 (mini scale).

**Step**	**Inlet-Air temperature [°C]**	**Exhaust-Air temperature [°C]**	**Drum-Speed [rpm]**	**Flow-Rate [m^3^/h]**	**Spray-Rate [g/min]**
**Warm up**	60	40	5	150	-
**Coating**	55	40	16	150	12
**Drying**	55	40	16	150	-
**Cooling**	25	30	5	150	-

**Table 3.** Coating parameters Pro-C-epT 4M8 (micro scale).

**Step**	**Inlet-Air temperature [°C]**	**Exhaust-Air temperature [°C]**	**Drum-Speed [rpm]**	**Flow-Rate [m^3^/h]**	**Spray-Rate [g/min]**
**Warm up**	60	43	3	18	-
**Coating**	55	43	14	18	1.83
**Drying**	55	43	14	18	-
**Cooling**	25	30	4	18	-

**Table 4.** Parameter set of the multivariate model.

**Model description**	**Results**
**Regression algorithm**	PLS
**Number of components**	3
**Calibration range**	0–12.7 mg
**Wavelength range**	1200–1400 cm^−1^
**R^2^ X**	0.944
**R^2^ Y**	0.994
**RMSEC (Mini scale)**	0.335
**RMSEP (Micro scale)**	0.392
